# Phenol-soluble modulin contributes to the dispersal of *Staphylococcus epidermidis* isolates from catheters

**DOI:** 10.3389/fmicb.2022.934358

**Published:** 2022-07-25

**Authors:** Yixin Jin, Qichen Wang, Haomin Zhang, Na Zhao, Ziyu Yang, Hua Wang, Min Li, Qian Liu

**Affiliations:** Department of Laboratory Medicine, Renji Hospital, Shanghai Jiao Tong University School of Medicine, Shanghai, China

**Keywords:** *Staphylococcus epidermidis*, catheter, sterile body fluids, biofilm, infection

## Abstract

Staphylococcus epidermidis (*S. epidermidis*), a human commensal, has been implicated in invasive infection in humans due to their ability to form biofilm. It is assumed that when a biofilm is dispersed it will subsequently cause a more severe infection. The clinical significance of *S. epidermidis* isolated from sterile body fluid (BF) remains unclear, and might be related to dispersal from catheter-associated biofilm infection. To evaluate this relationship, we evaluated *S. epidermidis* isolates from catheters (CA) or BF in hospitalized patients. Sequence type 2 (ST2) is the most prevalent type isolated from infection sites. Although the specific STs were also observed in isolates from different sites, we observed that the main sequence type was ST2, followed by ST59, among all the 114 isolates from different infection sites. Interestingly, ST2 strains isolated from BF exhibited significantly thicker biofilm than those from CA. The thicker biofilm was due to the higher expression of accumulation-associated protein (*aap*) but not intercellular adhesion (*ica*) operon. Moreover, the transcription of *PSM*δ and *PSM*ε were significantly increased in ST2 strains isolated from BF. Although the bacterial loads on catheters were similar infected by CA- or BF-originated strains in mouse biofilm-associated infection model, we observed a higher CFU in peri-catheter tissues infected by ST2 clones isolated from BF, suggesting that *S. epidermidis* with thicker biofilm formation might be able to disperse. Taken together, our data suggested that *S. epidermidis* originated from diverse infection sites exhibited different biofilm forming capacity. The major ST2 clone isolated from BF exhibited thicker biofilm by increasing the expression of Aap. The higher expression of PSM of these strains may contribute to bacteria dispersal from biofilm and the following bacterial spread.

## Introduction

The coagulase-negative Staphylococci, the major of which are *S. epidermidis*, are generally considered human commensals (Parlet et al., 2019; Liu et al., 2020; Otto, 2020). However, in certain situations, the microorganism is considered a human pathogen, due to its ability to form biofilms (Otto, 2009). About 40–90% of *S. epidermidis* infection is related to indwelling medical devices in critically ill or immune-compromised patients (Von Eiff et al., 2002). *S. epidermidis* is considered to be a contaminant when isolated from blood cultures in patients without implanted catheters or medical devices. However, when isolated from the blood stream in the presence of these foreign bodies or from BF, *S. epidermidis*’ role as a pathogen must be investigated (Shelburne et al., 2020).

The molecular epidemiology of *S. epidermidis* has been described extensively over the past several years. Although a considerable clonal heterogeneity of *S. epidermidis* has been observed, ST2 is generally considered a nosocomial sequence type (Cherifi et al., 2013; Du et al., 2013). Shelburne et al. (2020) reported that *S. epidermidis* ST2 is the main clone causing bloodstream infections by whole-genome sequencing analysis. Critically, these coagulase-negative staphylococci tend to be methicillin/oxacillin resistant when isolated from hospital setting (Lee et al., 2018).

Biofilm is reported to be the main virulence factor of *S. epidermidis* (Otto, 2013). The formation of biofilm includes three stages: primary adhesion, cell proliferation and aggregation, and separation (Otto, 2009). *S. epidermidis* produce many adhesins and virulence factors contributing to biofilm formation. AtlE promotes the initial adhesion by interacting with fibrinogen, fibronectin, and vitronectin in a non-covalent manner (Heilmann et al., 1997). Polysaccharide intercellular adhesin (PIA), which is produced and modified by *icaABCD* (intercellular adhesion ABCD) operon, is deemed as the key factor for aggregation of biofilm (Rohde et al., 2007). However, *ica*-negative *S. epidermidis* strains, isolated from clinical samples, can still produce strong biofilm (Rohde et al., 2007). Accumulation-associated protein (Aap) protein is mainly responsible for intercellular accumulation during biofilm formation (Hussain et al., 1997; Rohde et al., 2005; Sun et al., 2005). It was demonstrated that Aap is necessary for infection in a rat catheter model (Schaeffer et al., 2015).

The maturation and dispersal of biofilms are mainly regulated by the global regulatory quorum sensing system accessory gene regulator (Agr). Bacteria with *agr* dysfunction produce thicker biofilm and have obvious dispersion defects (Miragaia et al., 2005). It was reported that phenol-soluble modulins (PSMs), which are positively regulated by Agr, contribute to the dispersion of biofilms (Otto, 2004). *S. epidermidis* produces several PSMs, including α class PSMs and β class PSMs (Otto, 2014). α-type PSM such as PSMδ can lyse eukaryotic cells, including white blood cells, red blood cells, epithelial cells, endothelial cells, osteoblasts, etc. (Cheung et al., 2014). Although PSMs were deemed as virulence factors in *S. aureus*, PSMs produced by *S. epidermidis* are not involved in virulence (Queck et al., 2009). For example, PSMγ and PSMδ produced by the commensal *S. epidermidis* functionally cooperated with each other and LL-37 to enhance antimicrobial activity to *S. aureus* (Cogen et al., 2010).

Possession of mobile genetic element may confer selective advantage in invasive infection. To date, this has yet to be demonstrated in molecular epidemiologic evaluation involving *S. epidermidis* (Meric et al., 2018). Although it was reported that the catheter-related bacteria can spread and cause bloodstream infection (See et al., 2016), the connection of strains originated from different infection sites is still unknown. In the study, by analyzing the epidemiological characteristics of *S. epidermidis* from CA and sterile BF sites, we observed that the higher expression of PSM may contribute to the biofilm-related dispersal of bacteria.

## Materials and methods

### Ethics statement

The animal experiment was performed in accordance with the Guide for the Care and Use of Laboratory Animals. The animal protocol and the human samples collection were approved by the ethics committee of Renji Hospital, Shanghai Jiao Tong University School of Medicine. Blood were collected from the venous blood of healthy individuals. The written informed consents were received from all human subjects.

### Bacteria

*Staphylococcus epidermidis* isolates were collected from Renji hospital in Shanghai, China, over a 5-year period (2014–2018). Isolates recovered from sputum, urine, or wound samples were excluded to avoid the possible contamination. To avoid the possible interference of contaminated bacteria, *S. epidermidis* isolated from catheters (CA) and sterile body fluids (BF) were deemed as infection-originated strains, and included for the study. Here, sterile BF included: blood, pleural fluid, abdominal fluid, cerebrospinal fluid, bile, and drainage. We should mention that only strains with positive bacteria for bilateral double bottles for blood culture were enrolled and analyzed. All the bacteria used for the study were listed in Supplementary Table 1.

### Multilocus sequence typing

Multilocus sequence typing (MLST) was performed through amplification of the 7 housekeeping genes: *arc*, *aroE*, *gtr*, *mutS*, *pyrR*, *tpi*, and *yqiL* (Thomas et al., 2007). The primers for amplification of seven housekeeping genes were listed in Supplementary Table 2. The allelic numbers for PCR sequences were determined by the existing sequences available at the MLST website^1^.

### Antimicrobial susceptibility testing

*Staphylococcus epidermidis* isolates were tested for the following antibiotics: oxicillin (OXA), rifampicin (RF), co-trimoxazole (SXT), clindamycin (CLI), vancomycin, tigecycline, and linezolid using Vitek 2 Compact (Biomerieux). *S. aureus* ATCC29213 was used as a quality control.

### Semi-quantitative biofilm formation and primary attachment assay

Semi-quantitative biofilm formation and primary attachment assay was performed as described by Liu et al. (2011). For semi-quantitative biofilm formation, overnight cultures of *S. epidermidis* strains were diluted 1:100 into fresh tryptic soy broth (TSB). The diluted cultures were pipetted into sterile 96-well flat-bottom tissue culture plates and incubated at 37°C for 24 h.

For primary attachment assay, overnight cultures of *S. epidermidis* strains were diluted in fresh TSB and grown at 37°C with shaking (200 rpm) for 3 h. The bacteria were normalized to an OD_600_ of 1.0, and pipetted into sterile 96-well flat-bottom plate and incubated at 37°C for 1 h.

Culture supernatants were gently removed, and wells were gently washed twice with phosphate-buffered saline (PBS). The adherent organisms at the bottom of the wells were fixed by Bouin fixative over 1 h. After removing the fixative, the wells were washed twice with PBS and stained with 0.4% crystal violet, de-stained with slow-running water and dried at room temperature. The thickness of biofilm was qualified using absorbance with a MicroELISA autoreader (BioTek, Synergy 2) at 570 nm.

### Biofilm by confocal laser scanning microscopy

All ST2 isolates were used for CLSM. Briefly, overnight cultures of *S. epidermidis* strains were diluted 1:100 into fresh TSB with 0.5% glucose. The diluted cultures were pipetted into CysRabel-96 -well black bottom plate (CellCarrier) and incubated at 37°C for 48 h. After washing gently with PBS, the biofilms were fixed with paraformaldehyde (4%) and stained with propidium iodide (PI, 4 μM, red), and SYTO 9 (green) for 15 min. The biofilms were imaged using CLSM on a Leica TCS SP8 confocal microscope. Image analyses were performed using Image J.

### Hemolysis assay

*Staphylococcus epidermidis* were grown in TSB at 37°C, 200 rpm for 16 h, after which time culture supernatant (100 μl) were collected and incubated with 100 μl human red blood cells (2% [v/v] in PBS) in a 96-well round-bottom plate at 37°C for 1 h. Plates were centrifuged for 10 min at 1,500 rpm, the supernatant (100 μl) was collected and transferred into an additional sterile 96-well flat-bottom plate. The hemolytic activity was measured by testing OD_540_ using a Synerge 2 autoreader (BioTek).

### The carriage of virulence genes in *Staphylococcus epidermidis*

The presences of the following genes were tested by PCR for all the isolates: formate dehydrogenase (*fdh*), *mecA*, intercellular adhesion B (*icaB*), accumulation-associated protein (*aap*), autolysin (*atlE*) genes. The existence of mobile genetic element IS256 was also tested by PCR. The primers were listed in Supplementary Table 2.

### Real-time quantitative reverse transcription-PCR

The *S. epidermidis* strains were grown to stationary phase. The cells were centrifuged and the pellets were lysed with a FastPrep-96 (MP Biomedicals Products) at 800 rpm for 300 s three times. Supernatants were collected for total RNA isolation according to the manufacturer’s instructions (Qiagen). After DNase treatment with a TRUBO DNA-free kit (Ambion), 1 μg of total RNA was reverse transcribed with a Prime Script RT reagent kit (Qiagen). The cDNA was used as a template for RT-PCR with SYBR Green PCR reagents (Roche). Reactions were performed in a MicroAmp optical 96-well reaction plate with a 7500 sequence detector (Applied Biosystems).

### Mouse catheter-related infection model

A murine subcutaneous foreign body infection model was performed as described by Le et al. (2019). All ST2 *S. epidermidis* strains were grown at 37°C to the stationary phase in TSB. After centrifugation (4,000 rpm, 10 min), the bacteria pellets were washed with fresh TSB twice, and suspended in TSB with 0.5% glucose. One-centimeter long catheters were incubated with the bacteria suspension for 2 h at 37°C. The biofilm-coated catheters were gently washed with PBS and air-dried for 30 min. BALB/c female mice (4∼6 weeks old) were anesthetized with Avertin. Biofilm-coated catheters were implanted subcutaneously at the flank of the mouse. On the 4th day, the animals were sacrificed. Catheters, skin and soft tissues surrounding catheters, and the organs (livers, spleens, kidneys) were aseptically removed. The catheters were sonicated for 30 s in PBS. The tissues were homogenized in 500 μl PBS. The catheters, and homogenized tissues were diluted and plated on 5% sheep blood agar for CFU determination.

## Results

### Demographic characteristics

Totally, 114 *S. epidermidis* strains, isolated from CA (66 isolates, 57.9%) or BF (48 isolates, 42.1%), were used for the following experiments (Supplementary Table 1). There was no statistical difference in gender among the isolates from CA and BF. The isolates from CA group between 18 and 44 years were significantly higher than those from BF group (Table 1).

**TABLE 1:** Basic information of *Staphylococcus epidermidis* strains isolated from patients.

	Gender/Age	Origins	*P*-value
Gender (*n*, %)	Male (72, 63.2%)	CA (42, 58.3%)	0.0664
		BF (30, 41.7%)	
	Female (42, 36.8%)	CA (24, 57.1%)	0.2752
		BF (18, 42.9%)	
Age (*n*, %)	≤18 year (14, 12.3%)	CA (10, 71.4%)	0.0570
		BF (4. 28.6%)	
	18–44 years (24, 21.1%)	CA (16, 66.7.4%)	**0.0422**
		BF (8, 33.3%)	
	45–59 years (23, 20.2%)	CA (13, 56.5%)	0.5559
		BF (10, 43.5%)	
	≥60 year (53, 46.5%)	CA (27, 50.9%)	>0.9999
		BF (26, 49.1%)	

The statistical significance was measured by Chi-Square test. The bolded value means significant differences.

### The epidemiological characteristics of *Staphylococcus epidermidis* isolates

**FIGURE 1:**
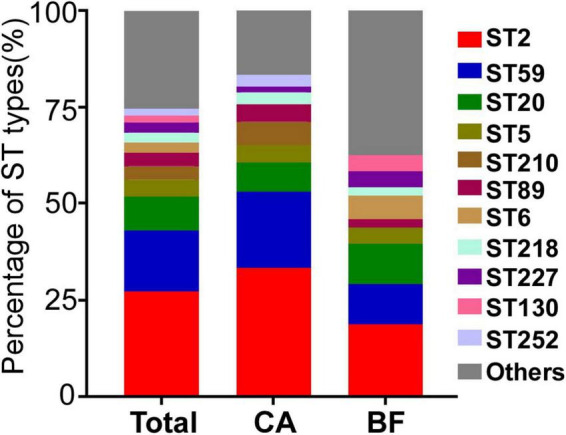
The MLST types of *Staphylococcus epidermidis* strains isolated from different infection sites. CA, isolates from catheters; BF, isolates from sterile body fluids.

The molecular epidemiological characteristics of the 114 *S. epidermidis* strains were examined. Totally, 38 STs were obtained. Among them, 13 STs were observed in both groups. 9 specific STs belonged to CA group, while 16 STs were existed only in BF group. ST2 was the predominant genotype in both groups (33.3% in CA group; 18.8% in BF group), followed by ST59 (19.7% in CA group; 10.4% in BF group) and ST20 (7.6% in CA group; 10.4% in BF group) (Figure 1 and Supplementary Table 1). Interestingly, although ST210 was the 4th most dominant sequence type in CA group, no ST210 type was found in the BF group. The similar distribution was observed for ST6, the 4th predominant sequence type in the BF group, which was also not found in CA group (Supplementary Table 1).

### Antibiotic resistance of *Staphylococcus epidermidis* isolates

**FIGURE 2:**
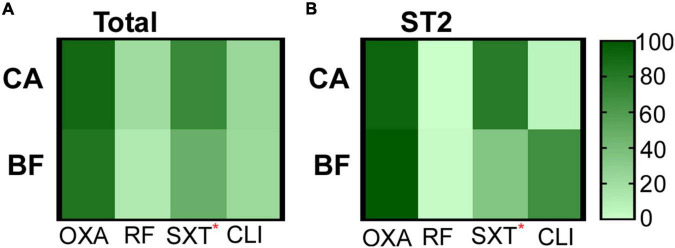
The antibiotic resistance of strains isolated from different groups. **(A)** All S. epidermidis isolates. **(B)** ST2 isolates. CA, isolates from catheters; BF, isolates from sterile body fluids; oxicillin (OXA), rifampicin (RF), co-trimoxazole (SXT), clindamycin (CLI). The statistical significance was measured by Chi-Square test. *p < 0.05.

Next, we examined the antibiotic resistance of the 114 *S. epidermidis* isolates to the commonly used antibiotics. All strains were sensitive to vancomycin, tigecycline and linezolid (data not shown). We observed that MRSE account for 87.7% (100/114) of all *S. epidermidis* isolates. Among them, 90.91% (60/66) MRSE were from CA group, while 83.33% (40/48) MRSE were from BF group (Figure 2A). The strains from CA group showed significantly higher resistance to co-trimoxazole (chi-square test, *P* = 0.012) compared with strains from BF group (Figure 2A).

The antibiotic resistances of the major clone ST2 were further compared. ST2 from CA group are more resistant to co-trimoxazole (chi-square test, *P* = 0.043) compared with the strains from BF group (Figure 2B).

### ST2 isolates from body fluid group exhibit thicker biofilm than strains From catheters group

**FIGURE 3:**
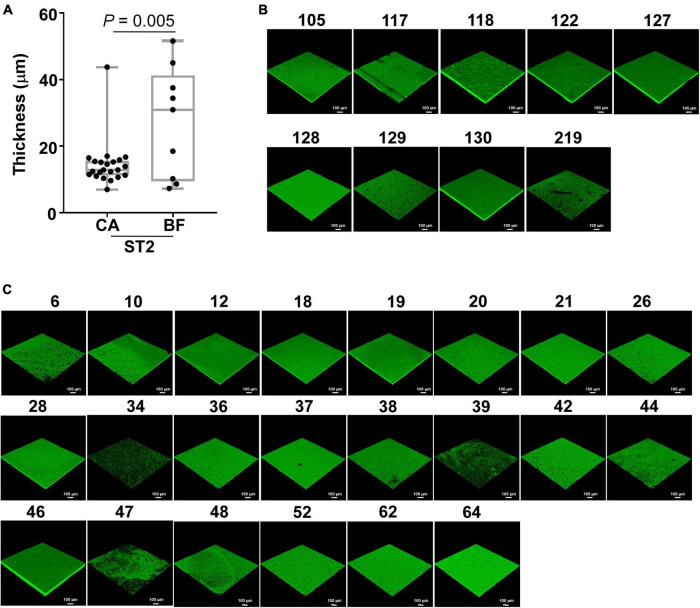
Biofilm formation of *Staphylococcus epidermidis* ST2 strains from different infection sites. Biofilms were grown in TSB with 0.5% glucose in static condition for 48 h. After gently washing and staining, the biofilms were observed by CLSM and analyzed by Image J. **(A)** Thickness of biofilm. **(B)** Representative CLSM example pictures for ST2 strains from BF group. **(C)** Representative CLSM example pictures for ST2 strains from CA group. The numbers on the pictures indicated the NO of clinical strains (Supplementary Table 1). The statistical significance was measured by unpaired, two-tailed *t*-test. Error bars show the mean ± SD.

Although ST2 was the predominant *S. epidermidis* type in both CA- and BF-group, isolates from CA group seemed to be more resistant to certain antibiotics, suggesting that the strains from different infection sites might have specific characteristics. Since biofilm formation contributes to antibiotic resistance for *S. epidermidis* (Otto, 2013), we compared the biofilm forming capability of the isolated strains by semi-quantitative biofilm assay. As shown, the biofilm forming capability was not significantly different among the strains isolated from CA and BF group (Supplementary Figure 1A). However, we observed that ST2 strains from CA group exhibited significantly weaker biofilm compared with those from BF group (Supplementary Figure 1B). To test the exact stage of biofilm formation, a primary attachment assay was tested for all ST2 clones. We observed that there are no differences of primary attachment of ST2 clones between the two groups (Supplementary Figure 1C). The static biofilms were further tested by CLSM. We observed significantly increased biofilm thickness of *S. epidermidis* ST2 strains isolated from BF group compared with CA group (Figure 3).

Because we observed thicker biofilm formation for ST2 isolated from BF, we hypothesized that the strains may exhibit different virulence which may cause diverse types of infection. Here, we tested this by detecting the hemolytic activity of all ST2 isolates. Interestingly, there are no differences of human erythrocytes-lysing ability of ST2 clones between the two groups (Supplementary Figure 1D).

### The biofilm-associated genes are highly expressed in ST2 isolated from body fluid

**FIGURE 4:**
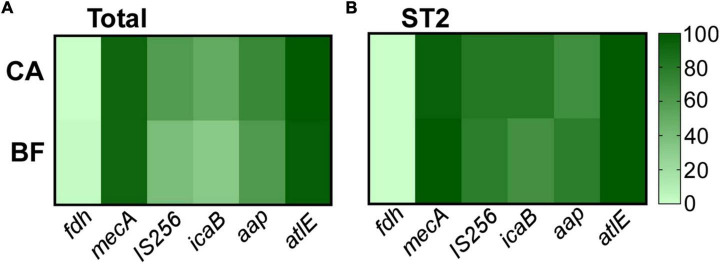
The content of virulence genes of *Staphylococcus epidermidis* isolates **(A)** and the major clone ST2 **(B)**. The gene content was determined by PCR.

We have observed significantly thicker biofilm in ST2 from BF group. To analyze whether the impact on biofilm formation is mediated by altering expression of biofilm-related genes, the transcriptional levels of several genes were compared. It was reported that the carriage of the genes were diverse in clinical isolates (Du et al., 2013), so the contents of genes were tested first. All the infection-originated strains did not carry formate dehydrogenase (*fdh*) gene, which is deemed as the biomarker for commensal (Conlan et al., 2012; Figure 4). Consistent with resistance to oxicillin, the carriage rates for *mecA* gene were over 90% for the isolates from each group (92.4% for CA group, 91.7% for BF group). Finally, we did not observe any differences of the carriage rates of insertion sequence 256 (IS256) and biofilm-related genes including *icaB*, *aap*, and *atlE* (Figure 4).

**FIGURE 5:**
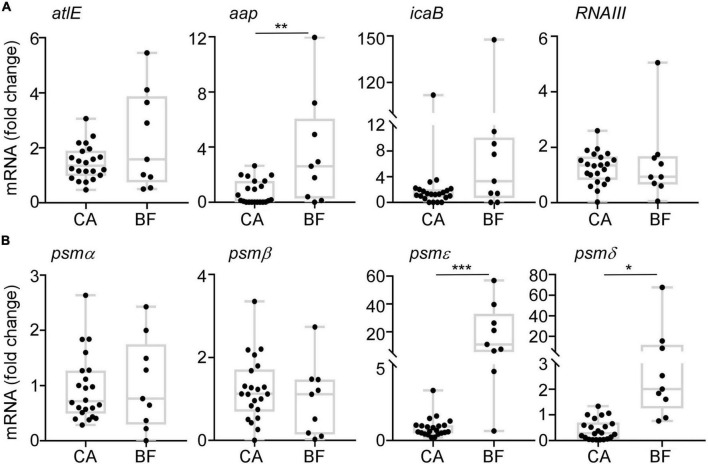
The transcription profiles of biofilm-associated gens. Cells were grown in TSB to stationary growth phase (OD_600_ = 2); then, the transcript levels were determined by qRT-PCR for *atlE*, *aap*, *icaB*, *RNAIII*, and *PSM* genes. The statistical significance was measured by unpaired, two-tailed *t*-test (*p < 0.05; **p < 0.01; ***p < 0.001).

Then, the transcription levels of biofilm-related genes were analyzed by quantitative RT-PCR. There is no significant differences on the expression of *atlE* gene (Figure 5A), which is associated with primary attachment (Heilmann et al., 1997). For the aggregation related genes including *aap* and *icaB*, we observed a significant increased transcription level of *aap* in ST2 isolates from BF group, which is consistent with the thicker biofilm formation (Figure 5A). We also tested the transcription of PSM, which is involved in the dispersal of biofilm (Otto, 2014). Interestingly, although there is no difference of *RNAIII* transcription between the two groups, the transcription levels of *PSM*ε and *PSM*δ were significantly higher in ST2 strains from BF group than from CA group (Figure 5B).

### The pathogenesis of *Staphylococcus epidermidis* clinical isolates using biofilm-associated infection model

To investigate whether the different biofilm formation of ST2 contribute to device-associated infection *in vivo*, we performed a murine subcutaneous foreign body infection model. The bacterial loads on the implanted catheters were measured. Meanwhile, the tissues around catheters and organs including liver, spleen, kidney were monitored to reflect the dissemination of the bacteria.

**FIGURE 6:**
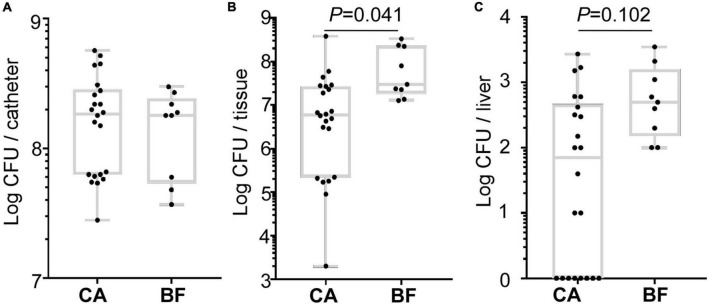
Comparison of the virulence of the *Staphylococcus epidermidis* isolates in a murine biofilm-associated infection model. CFU from catheters **(A)**, peri-catheter tissues **(B)** and livers **(C)** on day 4 post-infection. The statistical significance was measured by unpaired, two-tailed *t*-test. Error bars show the mean ± SD.

All ST2 isolates (22 strains from CA group, and 9 strains from BF group) were used for the *in vivo* assay. We observed that ST2 from both groups displayed similar bacterial loads on the catheters (Figure 6A). However, a significantly higher number of bacteria were found in peri-catheter tissues in the animals infected by ST2 from BF group, as compared with the animals infected by ST2 isolates from CA group (Figure 6B). Although bacteria were not recovered from spleens and kidneys (data not shown), a higher number of bacterial loads were also observed in the liver infected by the strains from BF group (Figure 6C). Taken together, our data indicated that ST2 with thicker biofilm forming capacity may tend to disseminate *S. epidermidis*.

## Discussion

*Staphylococcus epidermidis* is considered an opportunistic pathogen in human infections, particularly in bloodstream infections involving implanted catheters (Otto, 2013). Although the microorganism is thought to disperse to distal infection sites, to date, molecular epidemiology data linking isolates from implanted catheter with sterile BF have been limited. By comparing the molecular epidemiological characteristics of isolates from CA and BF, we demonstrated that ST2 is the predominant clones for all infection-related strains. Moreover, ST2 genotypes isolated from BF displayed significantly thicker biofilm than those from CA group. The BF-originated ST2 strains seemed to be prone to disperse to the tissues by up-regulating PSM.

The high heterogeneity of the *S. epidermidis* clones has been reported (Miragaia et al., 2002), we also observed a total of 38 clone types in 114 isolates. Among them, ST2 is the predominant type in both groups. ST2 was reported to be the most commonly identified clone type among *S. epidermidis* causing hospital-acquired infection (Ziebuhr et al., 2006; Li et al., 2009; Iorio et al., 2012; Widerstrom et al., 2012). By further analyzing the characteristics of ST2, we observed that the ST2 from BF group exhibited significantly thicker biofilm compared with those from catheters *in vitro* (Figure 3). Biofilm can be formed through polysaccharide and protein dependent pathways (Rohde et al., 2007). Here, we observed that the transcription of *icaB* is similar in isolates between CA and BF group. In contrast, the expression of *aap* was significantly higher in isolates from BF group (Figure 5), suggesting that the thicker biofilm for the isolates from BF group is mediated by biofilm-related proteins and not PIA.

The expression of PSMs is reported to be diverse in different *S. epidermidis* clinical isolates, for example, most of the infection-origin *S. epidermidis* failed to produce PSM (Vuong et al., 2004). PSMs are regulated by Agr system. To exclude the possibility that the different expression of PSM is due to the inactivation of Agr, we sequenced Agr operon for all ST2 isolates. Although several SNPs for AgrA and AgrC were observed for strains (Strain NO. 20, 38, 105, and 128 in Supplementary Table 1), the Agr activity is not affected for those strains by testing protease activity on skim-milk agar plates (data not shown). Bacteria will endure evolution during the infection by acquisition of mutations. For example, the virulence regulator Rsp was reported to evolve natural mutation in the host, which affects bacterial cytotoxicity and virulence in *S. aureus* (Das et al., 2016). Biofilm-associated *S. epidermidis* strain, which can cause bacteremia in patient, also endure in-host evolution about antibiotic resistance (Dengler Haunreiter et al., 2019). The limitation in our study is that the clinical isolates were from the different persons. The isolates backgrounds are variant due to the different host environment. Future study should focus on the strains isolated from different sites in the same patient. Although *PSM*β is reported to work as surfactant peptide which can promote biofilm dissemination in mice (Wang et al., 2011), our data showed that the transcription levels of α class PSMs (*PSM*ε and *PSM*δ) but not *PSM*β are significantly higher in BF-originated ST2 strains (Figure 5). PSM types may express inconsistently in different clinical isolates depending on the biological conditions.

As the main virulence factor of *S. epidermidis*, biofilm contributes to the antibiotic resistance (Otto, 2013). Our study showed that most of infection-originated strains are MRSE. Among all the ST2 clones, only 1 strain from CA group was MSSE. Oxacillin resistance is described in high proportions of *S. epidermidis* isolated from nosocomial settings. It might be that these isolates are endemic to the hospital environment. By tracing the medication history of patients, we observed that a total of 35 patients (20 from CA group, and 15 from BF group) accept antibiotics treatment (Supplementary Table 1). Whether the patient’s medication history contributes to the antibiotic resistance need to be further tested. Moreover, SCCmec cassette in MRSE encodes *PSM-mec*, which contributes to the pathogenesis of *S. epidermidis* sepsis (Qin et al., 2017). It is possible that the expression of *PSM-mec* in ST2 MRSE clinical isolatees contribute to the infection.

Taken together, our study confirmed that ST2 is the main clone type in the infection-originated *S. epidermidis* strains. Although it was assumed that bacteria causing severe infection in other organs are originated from catheters, isolates from CA and BF displayed different characteristics. BF-originated ST2 exhibited thicker biofilm forming capacity than those from catheters by increasing the Aap expression. The higher expression of PSM may contribute to the dispersal of ST2 with thicker biofilm.

## Data availability statement

The raw data supporting the conclusions of this article will be made available by the authors, without undue reservation.

## Ethics statement

The studies involving human participants were reviewed and approved by the Ethics Committee of Renji Hospital, Shanghai Jiao Tong University School of Medicine. The patients/participants provided their written informed consent to participate in this study.

## Author contributions

YJ and QW performed the experiments. HZ collected all the strains. NZ, ZY, and HW analyzed the data. HW, ML, and QL designed the study. YJ and QL wrote the manuscript. All authors approved the submitted version.

## Footnotes

1
http://www.mlst.net

## Supplementary material

Supplementary Figure 1 The general characteristics of *Staphylococcus epidermidis* from different infection sites. Biofilm formation by *S. epidermidis* for all isolates **(A)** and ST2 **(B)**. Biofilm formation was evaluated by a semi-quantitative biofilm assay using absorbance at 570 nm *in vitro*. **(C)** The primary attachment assay was tested by *S. epidermidis* for ST2 clones. **(D)** The hemolytic activity of *S. epidermidis* ST2 isolates. The results are representative of three independent experiments. The statistical significance was measured by unpaired, two-tailed *t*-test. Error bars show the mean ± SD.
