# Supplementary figures and images for: Phenol-soluble modulin contributes to the dispersal of Staphylococcus epidermidis isolates from catheters
Source: Front Microbiol. 2022 Jul 25;13:934358. doi: 10.3389/fmicb.2022.934358 (PMC9358717; doi:10.3389/fmicb.2022.934358)

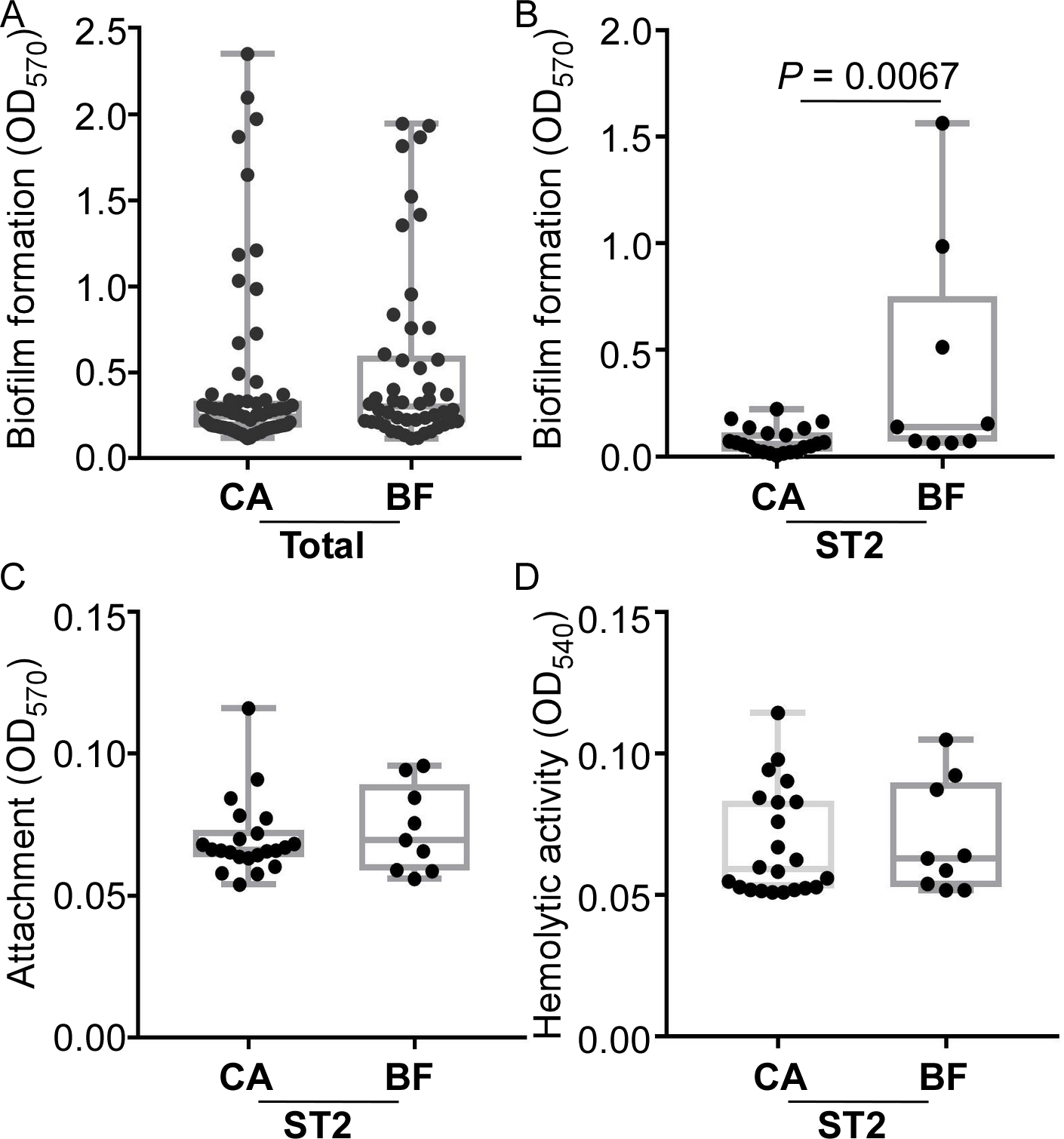

Supplement: Supplementary Figure 1 — The general characteristics of Staphylococcus epidermidis from different infection sites. Biofilm formation by S. epidermidis for all isolates (A) and ST2 (B). Biofilm formation was evaluated by a semi-quantitative biofilm assay using absorbance at 570 nm in vitro. (C) The primary attachment assay was tested by S. epidermidis for ST2 clones. (D) The hemolytic activity of S. epidermidis ST2 isolates. The results are representative of three independent experiments. The statistical significance was measured by unpaired, two-tailed t-test. Error bars show the mean ± SD. [file Image_1.TIF]
